# Targeting the *Xylella fastidiosa* spittlebug vector *Neophilaenus campestris* in the olive cover crops with the entomopathogenic fungus *Metarhizium brunneum*

**DOI:** 10.3389/finsc.2025.1579244

**Published:** 2025-04-21

**Authors:** Juan Carlos Conde-Bravo, María Fernández-Bravo, Inmaculada Garrido-Jurado, Meelad Yousef-Yousef, Enrique Quesada-Moraga

**Affiliations:** Laboratory of Agricultural Entomology, Department of Agronomy, María de Maeztu Excellence Unit DAUCO, ETSIAM, University of Cordoba, Cordoba, Spain

**Keywords:** *Philaenus spumarius*, pierce-sucking insects, aphrophoridae, biological control agent, olive quick decline syndrome

## Abstract

**Introduction:**

*Neophilaenus campestris* (Fallén) (Hemiptera: Aphrophoridae) is among the most abundant, highly dispersible, and widely distributed *Xylella fastidiosa* Wells (Xanthomonadales: Xanthomonadaceae) vectors to olive tree in Europe, with emphasis in Andalucía. The development of efficient and environmentally friendly vector management strategies is greatly needed. Entomopathogenic ascomycetes are among the few alternatives for the microbial control of pierce-sucking spittlebugs due to their unique contact mode and ability to endophytically colonize crops. These characteristics allow for several strategic uses aimed at reducing vector populations and/or their disease transmission potential. This study included a two-year field experiment to evaluate the *Metarhizium brunneum* Petch. (Ascomycota: Hypocreales) strain EAMa 01/58-Su sprayed onto *N. campestris* population naturally present in the olive grove cover in Cordoba (Spain).

**Methods:**

Experiments were conducted in early spring, and efficacy was evaluated using the Henderson-Tilton formula, as well as by analyzing changes in the relative population density of both nymphs and adults.

**Results and discussion:**

The fungus was detected in the soil and endophytically in the natural cover throughout the 8 days monitoring period, in which the fungal treatment significantly reduced both the nymph and the adult populations. Notably, the efficacy of the fungal treatment was 100.0% and 85.0% for foams and adults in 2023, and 62.5% and 72.0% for foams and adults in 2024, respectively. Results indicate a significant reduction in the population density of both vector developmental stages, highlighting the potential of this fungal strain for managing *X. fastidiosa* vectors in olive cover crops.

## 1 Introduction

Revealing today’s agricultural diseases caused by new pathogens that are increasingly resistant to pesticide treatments is a challenging task where the ecosystem plays an important role (1). Nevertheless, the evolution of non-sustainable agricultural activities and the removal of natural barriers due to intense human activity have reached such a level that only climatic factors can slow down the spread of new invasive species (2). This increasing spread of invasive species is further exacerbated by the growing homogenization of cultivated species worldwide (3), which reduces ecosystem resilience and facilitates the proliferation of concurrent pests and diseases (4). The *Xylella fastidiosa* Wells (Xanthomonadales: Xanthomonadaceae) bacterium has emerged as a serious threat to European agriculture after been the subspecies “pauca” first reported in Apulia, in Southern Italy causing olive quick decline syndrome in olive trees (5), and subsequent outbreaks of several subspecies and strains of *X. fastidiosa* in France and Spain (6–8). These problems are aggravated by the appearance of competent insect vectors, which transmit the pathogen to the cultivated plants (9). These insect vectors that feed on the plant’s xylem sap belong to the order Hemiptera (10) and are widely distributed. Within this group of insects, the suborder Cicadomorpha is identified as comprising the main insect vectors, mainly the families Cicadellidae (sharpshooters) and Aphrophoridae (spittlebugs) (11, 12). The latter is known as spittlebugs because of the secretion of protective foam over the nymph stage. Certain species of Aphrophoridae could pick up the pathogen from diseased plants and infect other plants (13, 14). In Europe, a wide range of these insect vectors are present (15). The main species to be considered are *Philaenus* sp*umarius* Linnaeus*, P. italosignus* Drosopoulos and Remane, and *Neophilaenus campestris* (Fallén) (16). As with most vector-borne pathogens, effective management of the vector is crucial for controlling the pathogen itself (17–21). Bodino et al. (22) clearly illustrate the phenological development of these insects, emphasizing that the nymph stage occurs within a limited period during spring. This underscores the importance of applying targeted treatments during this crucial phase as part of an Integrated Pest Management (IPM) strategy to prevent nymph migration into olive trees. The growing threat of vector-borne pathogens, coupled with the harmful effects of insecticides on humans and the environment, has spurred interest in sustainable pest control alternatives such as microbial control using entomopathogenic fungi (EPF). Several species such as *Hirsutella homalodiscae* nom. Prov., *Beauveria bassiana* Balsamo Vuill., *Isaria poprawskii* Caban., J.H, de Leon, Humber, K.D, Murray & W.A, Jones, *Metarhizium anisopliae* (Metschn.) Sorokin, *Trichothecium roseum* (Pers.) Link, and *Lecanicillium aphanocladii* Zare & W. Gams (Ascomycota: Hypocreales) have been identified as potential agents for regulating natural populations of American and European vectors of *X. fastidiosa* (8, 23–28). Hence, the use of entomopathogenic endophytic ascomycetes has become a crucial and highly valued component of today´s IPM programs, as there are already promising results against chewing and sap-sucking insect pests (8, 29–31). The application of the EPF *M. brunneum* Petch. EAMa 01/58-Su strain targeting olive fruit fly preimaginals in the soil beneath the tree canopy, either in autumn or spring, is currently a pre-commercial strategy. However, the impact of such treatments on other olive pests remains unexplored (32, 33). Likewise, the endophytic behavior of this fungal strain is an added value that could lead to the management of the natural and artificial cover olive crops under the tree or between lanes in which potential vectors of the bacterium are dwelling (34). Hereby, we have investigated the *M. brunneum* EAMa 01/58-Su strain-related strategic option of targeting a natural *N. campestris* nymph and adult populations in the cover crop of an olive grove in Cordoba (Spain) for the sustainable management of the vector-borne *X. fastidiosa*.

## 2 Materials and methods

### 2.1 Fungal strain

*Metarhizium brunneum* EAMa 01/58-Su strain was originally isolated from soil in a wheat plantation at Hinojosa del Duque, Cordoba (Spain) and deposited in the Spanish collection of culture types (CECT) with accession number CECT 20764. This strain was exclusively licensed by the University of Cordoba to KOPPERT B.V. in 2021. The fungal strain was produced in our laboratory on Petri plates with malt agar (MA) culture medium. It was incubated at 25 ± 1°C for 15 days in darkness, until fungal sporulation. The viability of conidia was determined to be above 95% before suspension preparation by germinating tests in MA medium. Conidia were mixed with sterile deionized water with Tween 80 (0.1% v/v) to obtain a conidial suspension with a final concentration of 1.0 × 10^8^ conidia/ml in the application tank. Before soil application, samples of the fungal suspension were directly collected from the nozzle of the sprayer to quantify the final concentration. Then, the conidial concentration was quantified with a Malassez chamber, resulting in a 1.5 × 10^7^ conidia/ml.

### 2.2 Experimental site and study design

The experiments were performed in an experimental olive orchard located at the Rabanales University Campus in Córdoba (37° 55’ 14.05’’ N, 4° 43’ 16.31’’ W; 137 masl) during spring in 2023 and 2024. A randomized block design was applied, with two treatments (fungal application and control) and four replicates each. The experimental unit of each block consisted of an area of 80 m^2^ (10 × 8 m). Considering that even if not been immotile Aphrophoridae nymphs only change the feeding place on the same plant (last nymph) or change the host plant with an adjacent one, there was a 30m distance among plots to avoid overlapping (35, 36). The fungal suspension was distributed throughout each treated area with a 14 liter capacity Stihl SR450 sprayer (Andreas Stihl S.A., Madrid, Spain), and sterile deionized water with Tween 80 (0.1% v/v) solution was used as control. A total of 3.5 liters of fungal suspension were distributed per block resulting in a concentration of 4.36 × 10^9^ conidia per square meter. The nymph vector population was estimated before the treatment (on the day of inoculation) and up to the 8th day post-inoculation. After fungal application, population recording was carried out every two days. According to estimates made in peripheral areas of the selected plots, the fourth instar of *N. campestris* was the most representative, given that the emergence of adults was recorded during the 8 days of monitoring. The time course evolution of the daily minimum, maximum, and mean temperatures, as well as relative humidity (RH), were recorded at the agro-climatic station of the Andalusian Institute for Agricultural, Fisheries, Food, and Organic Production Research and Training (IFAPA) in Alameda del Obispo (Cordoba). Rainfall was absent during both trial periods. The population was monitored using two different methods (37). On one side, the counting of foams produced by *N. campestris* nymphs, the primary targets of the treatment, was conducted on the adventitious plants in the olive grove. This was achieved using a circular frame with a 0.30 m diameter, randomly thrown five times within the area of each block and the foams counted (37). The focus on nymphs is particularly significant, as their limited mobility makes them more susceptible to localized treatments, thereby increasing the likelihood of effective control. In previous observations, the number of nymphs per foam was estimated between 1 and 1.5, depending on the species association of the arvense flora (38). On the other side, the capture of adults was evaluated using an entomological sleeve of 0.35 m diameter (37). Each sample consisted of ten entomological sleeving on the surface of the cover crop of each test block, with a total of three replicates per block. Captured adults were stored in a zip-lock plastic bag for later identification. To unravel the possible impact of the fungal spray, all alive insects captured with entomological sleeves during the trials underwent a selection process. Here, *N. campestris* insects were visually identified and separated from other insects using a 40× magnifying binocular. Then, selected insects were superficially disinfected by dipping them in a 5% sodium hypochlorite solution for 1 minute and then rinsed with sterile deionized water for 1 minute twice. Under sterile conditions, insects were placed in humid chambers, which consisted of a wet filter paper disc placed inside a Petri plate with 1 ml of sterile deionized water. All insect plates were incubated at 25 ± 1°C for 20 days. Subsequently, insects with fungal outgrowth on their surface were recorded.

### 2.3 Plant and soil sampling

On the day of inoculation (before treatment) and up to the 8th day post-inoculation, a sampling was performed to check the status of the presence and persistence of *Metarhizium* sp. in the soil. For each experimental block, 3 soil samples were randomly collected. Each sample was collected by extracting soil from the top 5 cm layer with a 10 cm diameter, using a small gardening shovel. Soil samples were then taken to the laboratory to be analyzed microbiologically. For that, one gram of soil was diluted in 9 ml of sterile deionized water with Tween 80 (0.1% v/v), and from that initial stock, five serial dilutions were obtained. All dilutions were seeded in Petri plates with Sabouraud Dextrose Agar (SDA) plus Dodine (65% p/p; 650g/kg) and incubated at 25 ± 1 °C for 15–20 days in darkness.

The spontaneous vegetation of the plots was very rich which was mainly composed of grasses such as *Bromus madritensis* (L.) (Poales: Poaceae), although the presence of other species such as *Calendula arvensis* (L.) (Asterales: Asteraceae), *Medicago arabica* (L.) (Fabales: Fabaceae), *Lolium rigidum* (G.) (Poales: Poaceae), *Brachypodium distachyon* (B.) (Poales: Poaceae) *Raphanus raphanistrum* (L.) (Brassicales: Brassicaceae), *Sinapis alba* (L.) (Brassicales: Brassicaceae) and *Vicia sativa* (L.) (Fabales: Fabaceae) could also be observed. Cover plant sampling was taken to represent the endophytic effect caused by the fungus. In each experimental block, three *B. madritensis* plant samples were randomly harvested at the end of the trial. The samples were stored in zip-lock plastic bags at 20°C until analysis in the laboratory one week later. The fungal re-isolation was carried out following the methods proposed by Raya-Díaz et al. (39), with some modifications. In this case, only the leaves of the plants were sampled. Fragments of 1cm lineal were placed on Petri plates with selective medium which consisted of 1l of sterile deionized water containing 65 g of sabouraud chloramphenicol dextrose agar, 15 g of agar, and 0.01 g of dodine.

### 2.4 Data analysis and statistics

The variation in relative population density (C) was calculated for each subplot according to Peveling et al. (40):


C= log(nf+1) − log(ni+1)

where “nf” is the number of viable insects at the end of the treatment and “ni” is the number of viable insects before treatment. Negative values of C indicate a decrease in relative population density and positive values an increase, compared to pre-treatment levels.

On the other side, the efficacy of the treatment was also calculated for each subplot according to Henderson and Tilton (41):


Efficacy (%) = 100 * [1−(Ti*Tratf/Tf * Trati)]

where T_i_ is the number of viable insects in the control before treatment, T_f_ is the number of viable insects in the control after 8 days from the treatment, Trat_i_ is the number of viable insects in the treated blocks before treatment and Trat_f_ is the number of viable insects in the treated blocks after 8 days from the treatment. The comparative analysis of efficiencies across the different subplots was conducted by examining the variation in the relative population density (C) values. The Kruskal-Wallis one-way non-parametric AOV was performed on the variation in relative population density (C) data from both treated and control fields using the All-Pairwise Comparisons of Mean Ranks test in the software Statistix 9.0 (Analytical Software, 2008).

The effect of the fungal application on the reduction of adult insects and nymphs of *N. campestris* over time in each year and their interactions were analyzed with a non-parametric generalized linear mixed model (GLMM) with a negative binomial distribution. This statistical approach was selected due to the significant overdispersion observed in the data. The analysis was performed using the *glmmTMB* package in R (42), which allows fitting mixed models with flexible distributions, such as the negative binomial and non-normal distributions. The fixed effects in the model included: i) treatments (treated *vs*. control), to evaluate the direct impact of the fungal application; ii) sampling date to capture temporal population trends; iii) year (2023 and 2024) to account for inter-annual variability; and iv) interactions between treatment, date, and year, to identify the potential of combined effects. Blocks were treated as a random effect to account for variability among experimental blocks and to prevent pseudoreplication. To assess the consistency of the treatment effects across years, a combined analysis was performed by including the interaction between treatment, date, and year in the model, allowing the evaluation of the cumulative or the interactive effects on adult and nymph populations over the years.

## 3 Results

### 3.1 Persistence of *M. brunneum* in the soil after treatment and endophytic detection in plants

**Figure 1:**
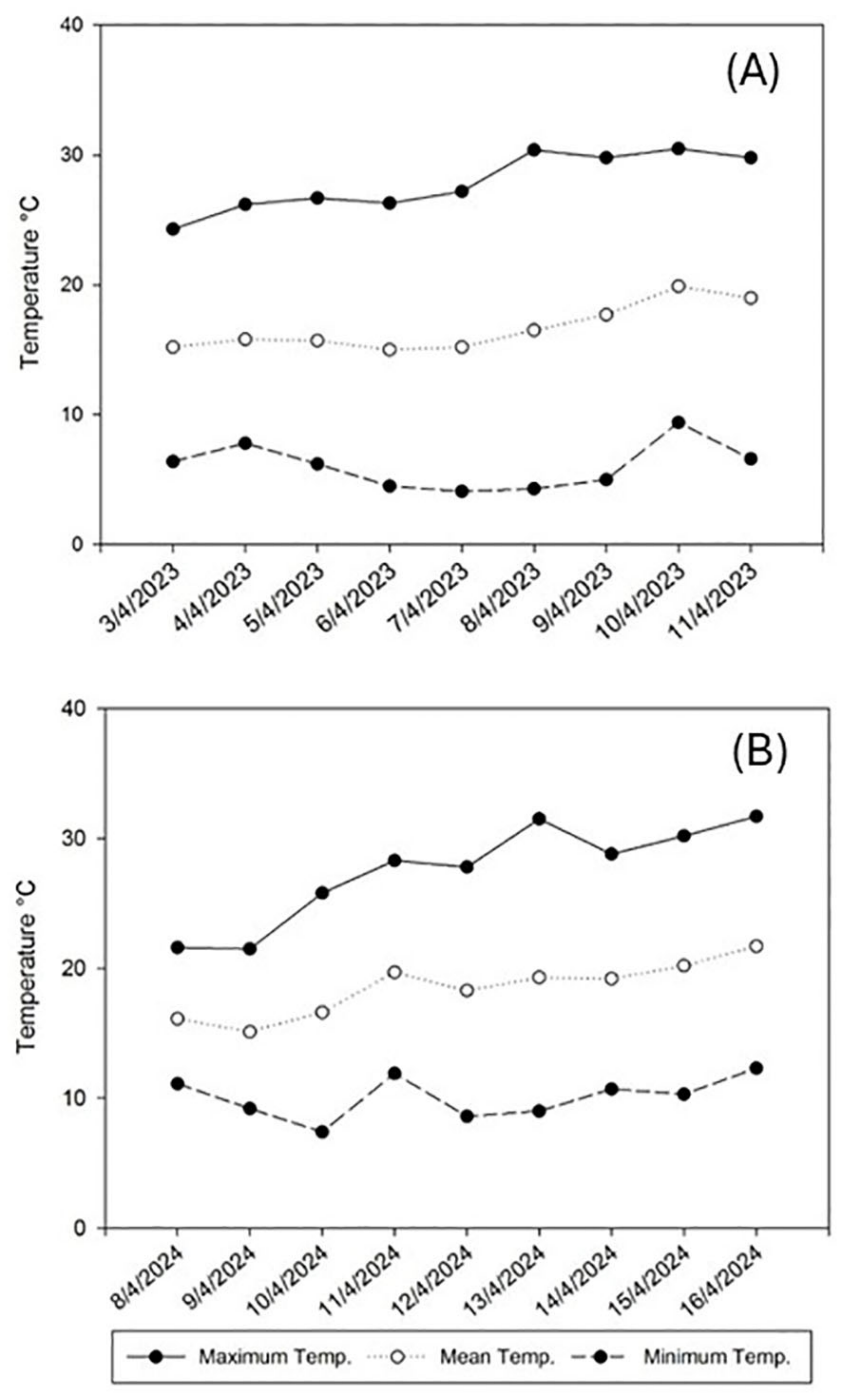
Temperature evolution in Alameda del Obispo (Cordoba) during the trial periods of spring 2023 **(A)** and spring 2024 **(B)**.

**Figure 2:**
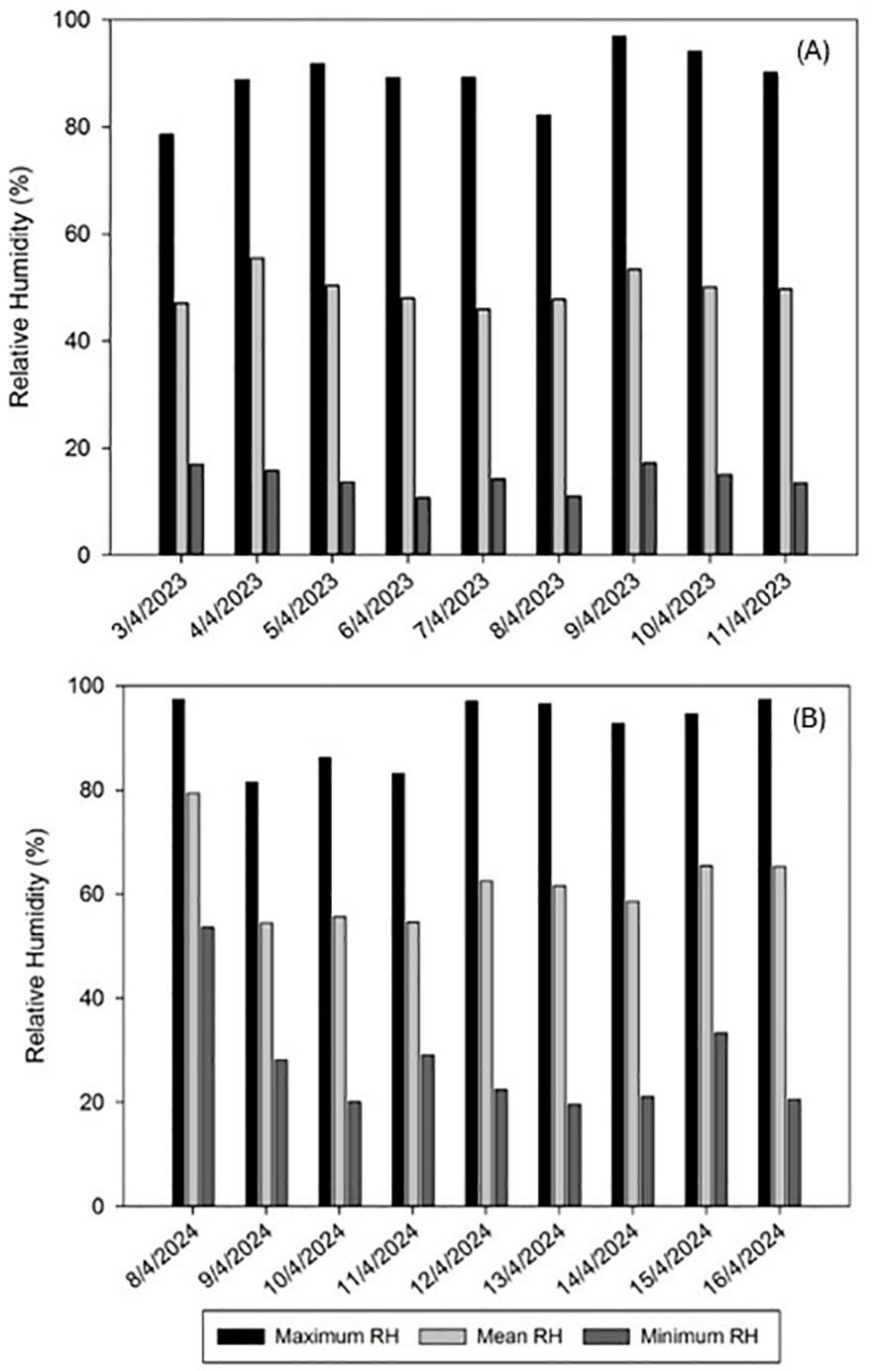
Relative humidity (RH) evolution in Alameda del Obispo (Cordoba) during the trial periods of spring 2023 **(A)** and spring 2024 **(B)**. No rainfall was recorded during both trial periods.

Considering the importance of the impact of climatological factors in a field trial, the average recorded temperature ranged between 15.0 and 19.9°C during 2023 and between 15.1 and 21.7°C in 2024, with the minimum and maximum temperatures ranging between 4.1 and 30.5°C and between 7.4 and 31.7°C in 2023 and 2024, respectively (**Figure 1**). The mean RH ranged between 46.0 and 55.5% during 2023 and between 54.5 and 79.4% in 2024, with the minimum and maximum RH ranging between 10.7 and 96.9% and between 19.7 and 97.3% in 2023 and 2024 respectively (**Figure 2**).

**Figure 3:**
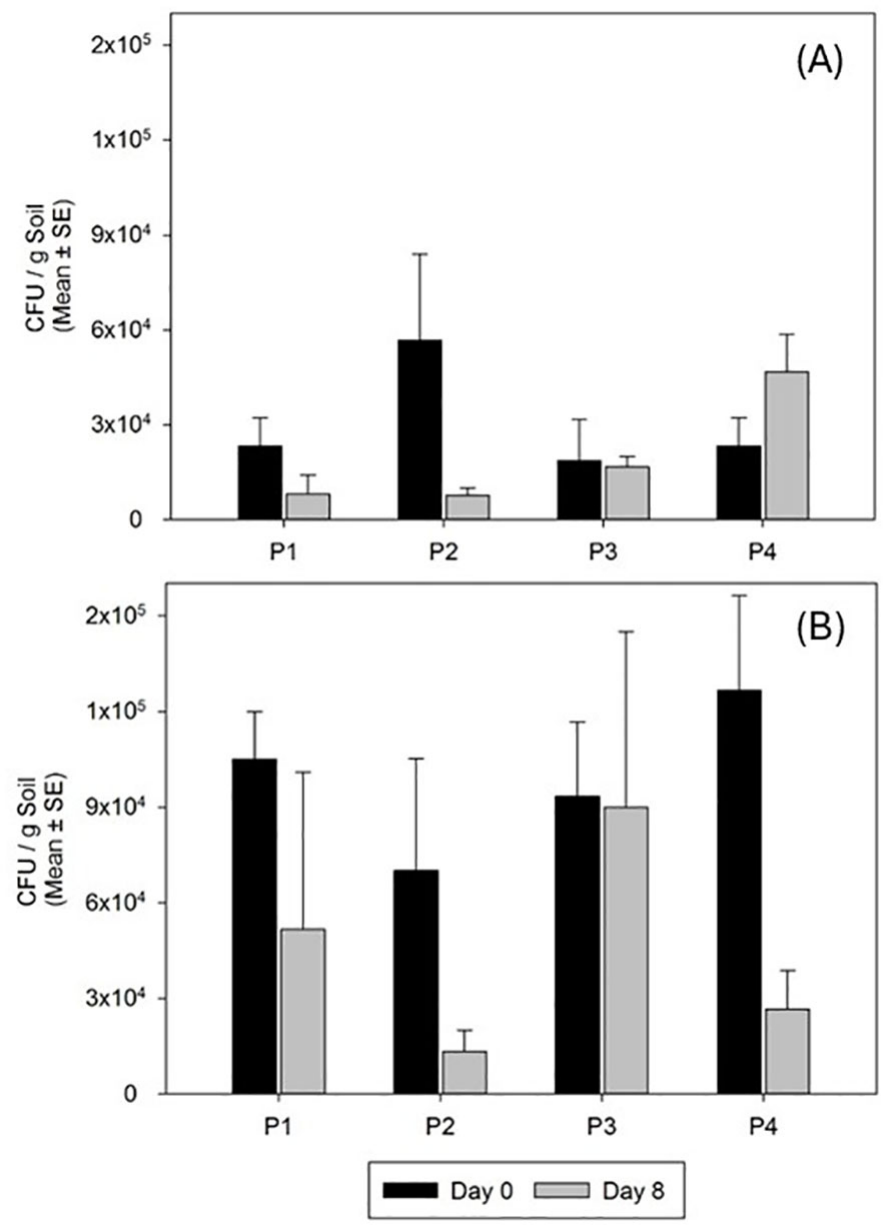
Persistence of *M. brunneum* EAMa 01/58-Su strain in the soil in spring 2023 **(A)** and spring 2024 **(B)**. P1, P2, P3, and P4 correspond to the subplots in the treated trial area. Day 0 shows CFU/g of soil just after fungal treatment and day 8 at the end of the trial.

Entomopathogenic fungus was not detected in any of the plants and soils of the control plots neither before nor after treatment, whereas it was endophytically detected in 38.0 ± 4.8% and 30.0 ± 10.7% of the cover plants in 2023 and 2024, respectively. In addition, the fungus was persistent in the soil during the sampling time after the treatments (**Figure 3**), with a constant concentration around 3.3 × 10^4^ conidia per gram of soil in the different subplots, in both replicates over time, and even with a steady increase at the end of the trial, as observed in subplot 4 (**Figure 3A**)

### 3.2 Evolution of *N. campestris* nymph and adult populations after spraying the cover crop with *M. brunneum*

**Figure 4:**
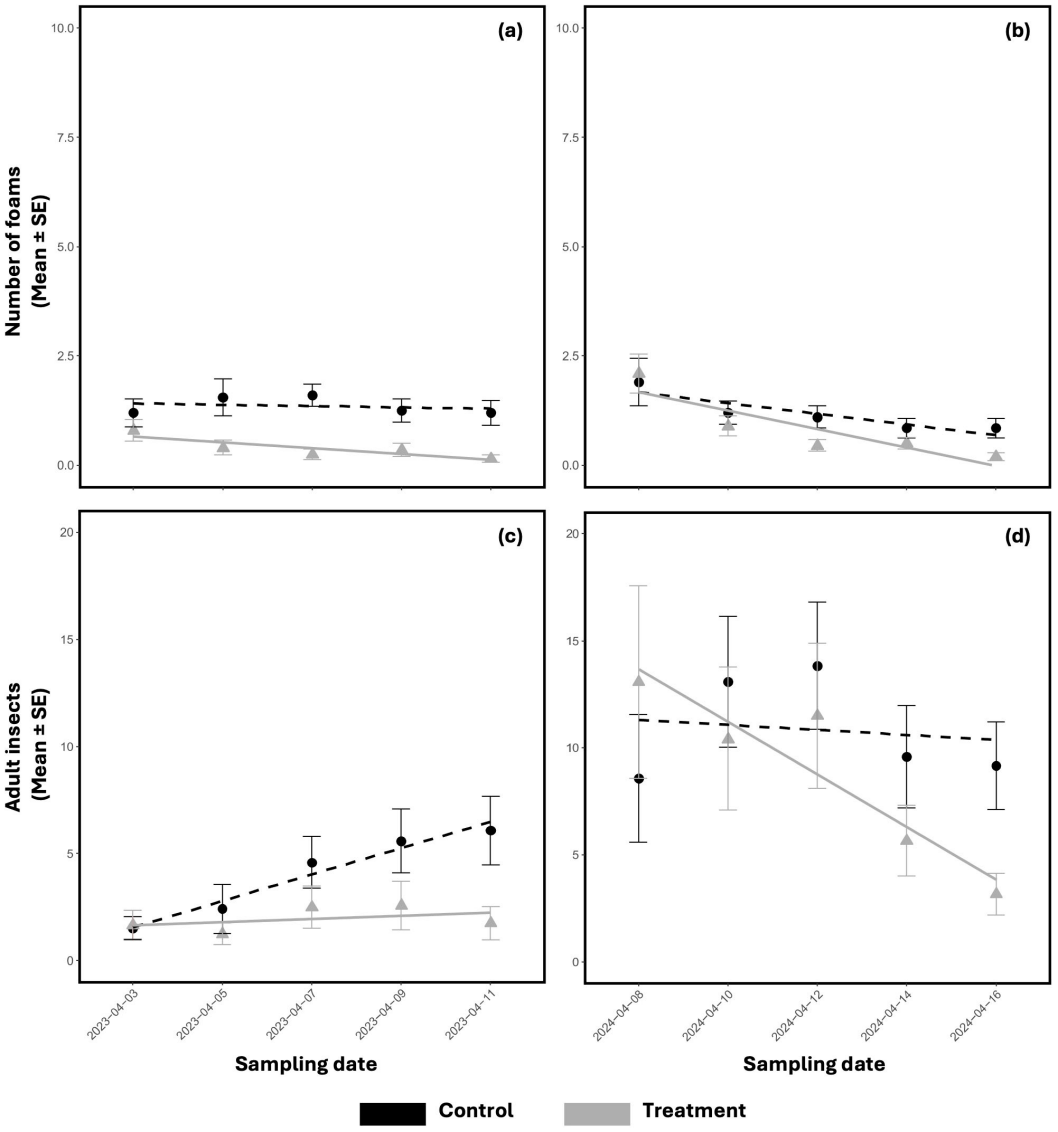
Evolution of the mean number ± SE of *N. campestris* foams counted in spring 2023 **(A)** and spring 2024 **(B)** and of the adults captured in spring 2023 **(C)** and spring 2024 **(D)** during the test period after spraying with *M. brunneum* EAMa 01/58-Su strain. Lines represent the tendency of nymph and adult populations per block along the time of the sampling date. Black lines with circles represent controls and grey lines with triangles represent treatments. Fungi were applied in treatment plots on the first day of sampling in each year and treatment blocks.

Considering the hierarchical structure of the experimental design, along with the data on adult insect captures and foam counts where nymphs reside, the trials conducted in 2023 and 2024 demonstrated that the application of *M. brunneum* strain EAMa 01/58-Su effectively reduced the population of *N. campestris* naturally present on the adventitious plants in the olive grove (**Figure 4**).

In 2023, a significant reduction of foams was observed in treated plots compared to the control along the time (β=−1.283, SE=0.512, p=0.013; **Supplementary Table S1**; **Figure 4A**). The mean number of foams recorded in control plots at the end of the experiment was 1.2 foams, while treated plots showed a mean of 0.2 foams, which reflected an 87.5% reduction. In 2024, there was a reduction of foams, but less pronounced and not statistically significant (β=−0.470, SE=0.492, p=0.314; **Supplementary Table S2**; **Figure 4B**). Nevertheless, the mean foam count in control plots at the end of the experiment was 0.9, compared to a mean number of 0.2 foams in treated plots, reflecting a 76.4% of reduction. Hence, from the combined analysis incorporating treatment, date, and year as interacting factors, no significant cumulative or interactive effects were detected for foams recorded between the two years (t=−0.430, p=0.665).

In 2023, a reduction in the treated adult population time was detected, compared to the control. Still, it was not statistically significant (β=−0.663, SE=0.372, p=0.078; **Supplementary Table S3**; **Figure 4C**). Similarly for foams, the mean capture in the control plots at the end of the experiment was 6.08 adults, compared to 1.8 in treated plots, representing a 71.2% reduction. In 2024, although a decrease in the adult population was observed in treated plots compared to the controls, it was not statistically significant (β=−0.301, SE=0.335, p=0.368; **Supplementary Table S4**; **Figure 4D**). The mean capture in the control plots at the end of the experiment was 9.2, while in treated plots was 3.2, representing an 83.7% reduction. When comparing adults decrease across both years, the combined analysis showed no significant effects of treatment or its interaction with date and year (t=−1.030, p=0.304), suggesting no clear cumulative or interactive effects on adult populations.

**Figure 5:**
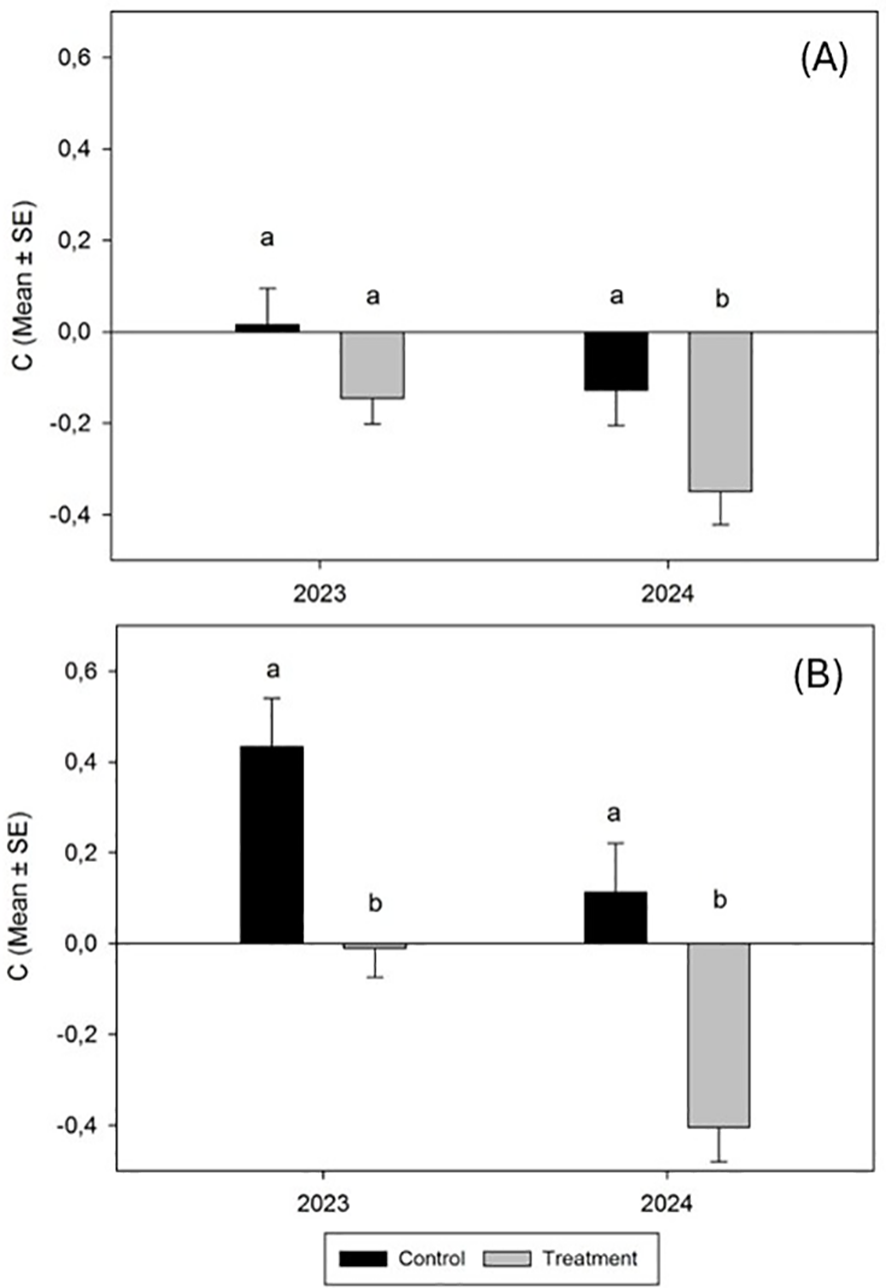
Variation in the relative population density (mean ± SE) of *N. campestris* foams **(A)** and adult insects **(B)** counted in spring 2023 and spring 2024 during the trial period after spraying the cover crop with *M. brunneum* EAMa 01/58-Su strain. This indicator is calculated according to Peveling et al. (40), where the viable population before and after the trial is considered. Negative values of C indicate a decrease in the trial population. Values not sharing the same letter are significantly different at p ≤ 0.05 (nonparametric Kruskal-Wallis test; alpha=0.05).

Conversely, the results of the variation of relative population density (C) after fungal application revealed that both the *N. campestris* foams counted and adult populations decreased during both trials. Meanwhile, the decrease did not reach significance for foams counted in the 2023 trial and 2024 (H = 1.8509, P < 0.1737; H = 3.3827, P < 0.0651 respectively) (**Figure 5A**). Nevertheless, the results of the variation of C exposed for *N. campestris* adults showed significant differences in both trials, 2023 and 2024 (H = 8.4397, P < 0.0017; H = 10.5082, P < 0.0012, respectively) (**Figure 5B**).

Whilst the efficacy of the fungal spray varied among subplots for each season, in the 2023 trial there was a 100.0% efficacy against foams for all the subplots, whereas in 2024, the treatment was proved to be highly effective (75–100% efficacy) in three subplots, with no significant impact in subplot 4 (**Table 1**). For adults, it reached moderate to high values, 66.7–100.0% and 33.3–98.8% in 2023 and 2024, respectively. Noteworthy, the efficacy of fungal treatment produced 2.06% and 1.63% of colonized insects after incubation in humid chambers during spring 2023 and 2024 respectively.

**Table 1:** Efficacy of *M. brunneum* fungal against *N. campestris*.

Stage	Subplot	Trials*	
		2023	2024
Foams	1	100.0%	75.0%
	2	100.0%	100.0%
	3	100.0%	75.0%
	4	100.0%	0.0%
Adults	1	100.0%	92.9%
	2	100.0%	63.1%
	3	73.3%	98.8%
	4	66.7%	33.3%

*Comparative effectiveness of *M. brunneum* EAMa 01/58-Su strain against foams and adult stages of *N. campestris* on olive cover crops in spring 2023 and 2024.

The efficacy factor calculation represents the comparison of the viable population before and after the trial between the control and the fungal treatment, according to Henderson and Tilton (41).

## 4 Discussion

Entomopathogenic fungi (EPF) are recognized as a crucial component of pest biological control strategies, as they help maintain the natural balance of insect populations. By reducing the reliance on continuous chemical applications, EPF contributes to promoting ecosystem sustainability. The application of microbial control agents is not widespread in agriculture, and the introduction of the non-curable pathogen *X. fastidiosa* to new countries such as Italy and Spain have led to the development of responsible management practices to control the vectors responsible for spreading the pathogen across Europe (4). Some Auchenorrhyncha insects such as *P.* sp*umarius*, *N. campestris* or *E. lineolatus* have been identified for acquiring *X. fastidiosa* bacteria (43). Likewise, the ability of *P.* sp*umarius* to transmit the bacterium between olive plants has also been studied (44). These insects are responsible for contributing to the movement of the bacteria to other healthy agronomic areas producing the olive quick decline syndrome (OQDS), so it is crucial to implement effective control measures for its management. To date, synthetic pyrethroids (deltamethrin and λ**-**cyhalothrin) and neonicotinoids (imidacloprid, acetamiprid, and thiamethoxam) have been highly effective against *P.* sp*umarius* (45–48). Deltamethrin and λ-cyhalothrin have exhibited results with mortalities over 86% after 24 h of exposure, which has maintained even after 72 h (47). Meanwhile, neonicotinoid-based insecticides are effective against nymph and adult sharpshooters (49). On the other side, Sulfoxaflor (Closer^®^) applied at a concentration of 64 ppm, showed comparable efficacy to deltamethrin under greenhouse conditions, achieving a 95% reduction of insects on plants compared to controls after 24 hours of exposure (47). However, the strict regulations on the use of synthetic pesticides in Europe have created a growing need for environmentally safer alternative management strategies for the vector control of *Xylella fastidiosa* (50, 51). EPF have been reported to naturally regulate insect pest populations and their inoculative and inundative use may help reduce the use of synthetic insecticides in the environment (52). Today, there are fascinating results from the application of the entomopathogenic fungus such as *Metarhizium* sp. soil application under the olive canopy for the control of third instar larvae of the olive fruit fly which fall to the ground to pupate in the autumn season, and before the emergence of adults in the spring, the fungal treatment has provided between 50 and 90% of reduction in *Bactrocera oleae* Rossi (Diptera: Tephritidae) population density (32, 33). Like the fungal species described above, the potential of versatile fungi such as *Trichoderma* sp. Pers. (Ascomycota: Hypocreales) have been considered biological control agents and have even been shown to have potential against *P.* sp*umarius* (53). Like this, *L. aphanocladii* and *B. bassiana* fungi have been successfully evaluated after direct spraying of conidia on nymphal stages of *P.* sp*umarius* showing mortalities ranging from 43 to 46% (8). In addition, *B. bassiana* strain BbGEp1 positively increased the mortality on *N. campestris* insects according to the concentration ranged between 10 and 80% with a median value lethal concentration of 1.61 × 10^6^ conidia/mL when the fungus is sprayed on to *Bromus hordeaceum* (54). Under microcosmos conditions, several commercially available mycoinsecticides have been tested against *P.* sp*umarius* nymphs, yielding variable results ranging from no activity to moderate or high efficacy (8). However, to date, no field evaluations have been conducted.

The present experimental study represents the first field control work against the natural occurrence of foams where nymphs dwell and adults of *N. campestris* with the application of the fungus *M. brunneum* EAMa 01/58-Su strain with very promising results. This strain demonstrated high virulence against both adults and foams of *N. campestris*, even at high population densities. This was evident when compared to standard population values previously reported in herbaceous olive covers in Apulia (Italy), which typically reach up to 0.25 individuals per sweep, though they rarely exceed 0.1 individuals per sweep (55). Efficacy of fungal application ranged from 66.7 to 100.0% and 33.3 to 98.8% for adults in the spring 2023 and 2024 trials respectively. On the other side, there was a 100.0% efficacy against foams for all the subplots of the spring 2023 trial. Nonetheless, efficacy remained above 75% in three subplots of the spring 2024 trial. Meanwhile, subplot 4 did not show efficacy as the population was not homogeneous in all subplots, so the limited number of insects in the subplot limited the treatment evaluation.

The presence of the fungus applied after wet-chambering was confirmed in 2.1% and 1.6% of the total adult insects captured during the spring 2023 and 2024 trial respectively. Some authors have highlighted that the virulence of native strains of EPF may surpass that of commercial strains, but their efficacy can be largely influenced by genetic variability and their adaptation to host-related environmental factors (56–58). In this sense, a selection of these factors such as humidity, temperature, and organic matter, among others, have been reported as key factors that affect *Metarhizium* spp. presence and persistence in soils (62). Thus, selecting environmentally competent fungal strains capable of persisting in the host environment throughout the required infection period is crucial (52). Meanwhile, adults in the control treatment exhibited a generalized progressive population increase, in contrast to the fungus-treated area, where the population trend declined. Similarly, the effects of endophytic EPF, including *Metarhizium* sp., on plant performance has become a focus of extensive research since the discovery of their relationship with a beneficial effect on plants (59, 60). In this work, plant samples collected of the cover crop were colonized with the endophytic ranging to 38.0 ± 4.8% and 30.0 ± 10.7% in 2023 and 2024 respectively. The fungus-insect contact via endophyte through its feeding on adventitious flora could be an added value on the double effect on the mortality of the *N. campestris* population and could explain the high efficacy of the fungal spray towards the adults coming from challenged nymphs.

The non-parametric generalized linear mixed-effects model analysis demonstrated that the application of *M. brunneum* EAMa 01/58-Su strain effectively reduced the population of *N. campestris* (both adults and foams) over the days evaluated following the fungal treatment compared to the controls in each year. Both, the differences in temperature and humidity conditions between 2023 and 2024 lead to more advanced developmental stages of the *N. campestris* population in the second year at the same date of the fungal treatment, and the fact that the contagious nymph distribution (35) lead to an initial population in the control plots somewhat higher than in the treated plots in 2023, could explain the observed differences in treatment efficacy among the two seasons, with adults showing more pronounced effects in 2023 and foams demonstrating stronger trends in 2023 than in 2024. The combined analysis of both years, for both adults and foams, demonstrated the robustness of the experimental repetitions, with no significant cumulative or interactive effects detected, highlighting consistent treatment impacts across years and the influence of natural population variability. Moreover, considering that the average number of nymphs per foam ranges between 1.5 and 2.0 (38, 61), it can be concluded that the adult captures in the plots during the experiment originated from nymphs that survived the treatment, with no evidence of invasion from neighboring plots.

Considering that the selected *M. brunneum* strain has been previously reported to have ecological fitness in Mediterranean agroecosystems (62), the strategy of targeting *X. fastidiosa* vector populations in the olive cover crops with the EAMa 01/58-Su strain could address the present European objectives for deploying environmentally friendly strategies for managing olive quick decline syndrome.

## 5 Conclusions

The fungal treatment demonstrated greater efficacy in reducing populations in 2023, particularly of foams, while the impact was less pronounced in 2024, with natural population trends and inter-annual variability likely influencing the observed results. In addition, it has also been scientifically proven that the fungus persisted in the soil during the sampling time. These results suggest that the soil application of *M. brunneum* EAMa 01/58-Su strain could be adopted as a potential biological control strategy within an IPM program to manage natural populations of *N. campestris*, one of the main vectors of *Xylella fastidiosa* in the Mediterranean basin. However, further future research including the evaluation of the compatibility between *M. brunneum* EAMa 01/58-Su strain and other management tools, such as cover crops, could improve the efficiency and sustainability of its use in the management of *X. fastidiosa*.

## Data Availability

The original contributions presented in the study are included in the article/**Supplementary Material**. Further inquiries can be directed to the corresponding author.
