# Supplementary material for: Targeting the Xylella fastidiosa spittlebug vector Neophilaenus campestris in the olive cover crops with the entomopathogenic fungus Metarhizium brunneum
Source: Front Insect Sci. 2025 Apr 21;5:1579244. doi: 10.3389/finsc.2025.1579244 (PMC12051512; doi:10.3389/finsc.2025.1579244)
Supplement: Supplementary file 1 [file Table1.docx]

**SUPPLEMENTARY MATERIAL TABLE S1** | Evolution of number of *N. campestris* foams counted in spring 2023 during the test period after spraying with *M. brunneum* EAMa 01/58-Su strain. Fungi were applied in treatment plots on the first day of sampling in each year and treatment blocks.

|  |  | Sampling Date | 03/04/2023 | 05/04/2023 | 07/04/2023 | 09/04/2023 | 11/04/2023 |
| --- | --- | --- | --- | --- | --- | --- | --- |
| TREATMENT | BLOCK 1 | S 1 | 0 | 1 | 0 | 0 | 0 |
|  |  | S 2 | 4 | 1 | 2 | 2 | 1 |
|  |  | S 3 | 2 | 3 | 0 | 2 | 0 |
|  |  | S 4 | 0 | 0 | 0 | 0 | 0 |
|  |  | S 5 | 2 | 1 | 0 | 0 | 0 |
|  |  | **Mean** | 1,6 | 1,2 | 0,4 | 0,8 | 0,2 |
|  | BLOCK 2 | S 1 | 0 | 0 | 0 | 0 | 0 |
|  |  | S 2 | 0 | 0 | 0 | 0 | 0 |
|  |  | S 3 | 0 | 0 | 0 | 0 | 0 |
|  |  | S 4 | 1 | 1 | 0 | 1 | 0 |
|  |  | S 5 | 1 | 0 | 1 | 0 | 0 |
|  |  | **Mean** | 0,4 | 0,2 | 0,2 | 0,2 | 0 |
|  | BLOCK 3 | S 1 | 0 | 0 | 0 | 0 | 0 |
|  |  | S 2 | 0 | 0 | 0 | 0 | 1 |
|  |  | S 3 | 2 | 0 | 0 | 1 | 0 |
|  |  | S 4 | 0 | 0 | 0 | 0 | 0 |
|  |  | S 5 | 0 | 0 | 0 | 0 | 0 |
|  |  | **Mean** | 0,4 | 0 | 0 | 0,2 | 0,2 |
|  | BLOCK 4 | S 1 | 1 | 1 | 0 | 0 | 0 |
|  |  | S 2 | 0 | 0 | 1 | 0 | 0 |
|  |  | S 3 | 2 | 0 | 0 | 1 | 0 |
|  |  | S 4 | 0 | 0 | 0 | 0 | 1 |
|  |  | S 5 | 1 | 0 | 1 | 0 | 0 |
|  |  | **Mean** | 0,8 | 0,2 | 0,4 | 0,2 | 0,2 |
| CONTROL | BLOCK 1 | S 1 | 0 | 1 | 2 | 1 | 1 |
|  |  | S 2 | 1 | 1 | 2 | 3 | 0 |
|  |  | S 3 | 0 | 4 | 0 | 0 | 0 |
|  |  | S 4 | 3 | 0 | 1 | 0 | 2 |
|  |  | S 5 | 2 | 0 | 3 | 1 | 3 |
|  |  | **Mean** | 1,2 | 1,2 | 1,6 | 1 | 1,2 |
|  | BLOCK 2 | S 1 | 2 | 0 | 1 | 0 | 0 |
|  |  | S 2 | 0 | 7 | 2 | 1 | 0 |
|  |  | S 3 | 0 | 0 | 0 | 1 | 4 |
|  |  | S 4 | 1 | 3 | 3 | 2 | 1 |
|  |  | S 5 | 5 | 3 | 3 | 4 | 1 |
|  |  | **Mean** | 1,6 | 2,6 | 1,8 | 1,6 | 1,2 |
|  | BLOCK 3 | S 1 | 0 | 0 | 3 | 1 | 0 |
|  |  | S 2 | 0 | 1 | 2 | 0 | 4 |
|  |  | S 3 | 2 | 1 | 1 | 0 | 1 |
|  |  | S 4 | 0 | 2 | 4 | 3 | 0 |
|  |  | S 5 | 3 | 0 | 1 | 2 | 2 |
|  |  | **Mean** | 1 | 0,8 | 2,2 | 1,2 | 1,4 |
|  | BLOCK 4 | S 1 | 0 | 2 | 1 | 2 | 1 |
|  |  | S 2 | 1 | 4 | 1 | 0 | 1 |
|  |  | S 3 | 1 | 2 | 1 | 2 | 1 |
|  |  | S 4 | 0 | 0 | 0 | 0 | 2 |
|  |  | S 5 | 3 | 0 | 1 | 2 | 0 |
|  |  | **Mean** | 1 | 1,6 | 0,8 | 1,2 | 1 |

**SUPPLEMENTARY MATERIAL TABLE S2** | Evolution of number of *N. campestris* foams counted in spring 2024 during the test period after spraying with *M. brunneum* EAMa 01/58-Su strain. Fungi were applied in treatment plots on the first day of sampling in each year and treatment blocks.

|  |  | Sampling Date | 08/04/2024 | 10/04/2024 | 12/04/2024 | 14/04/2024 | 16/04/2024 |
| --- | --- | --- | --- | --- | --- | --- | --- |
| TREATMENT | BLOCK 1 | S1 | 1 | 0 | 0 | 1 | 0 |
|  |  | S2 | 0 | 2 | 1 | 2 | 0 |
|  |  | S3 | 3 | 1 | 1 | 0 | 0 |
|  |  | S4 | 2 | 3 | 0 | 0 | 1 |
|  |  | S5 | 2 | 0 | 0 | 0 | 0 |
|  |  | **Mean** | 1,6 | 1,2 | 0,4 | 0,6 | 0,2 |
|  | BLOCK 2 | S1 | 4 | 1 | 1 | 0 | 0 |
|  |  | S2 | 1 | 0 | 1 | 1 | 0 |
|  |  | S3 | 4 | 0 | 1 | 0 | 0 |
|  |  | S4 | 4 | 1 | 2 | 0 | 0 |
|  |  | S5 | 8 | 3 | 0 | 1 | 0 |
|  |  | **Mean** | 4,2 | 1 | 1 | 0,4 | 0 |
|  | BLOCK 3 | S1 | 2 | 1 | 0 | 0 | 1 |
|  |  | S2 | 0 | 0 | 0 | 0 | 0 |
|  |  | S3 | 1 | 0 | 0 | 1 | 0 |
|  |  | S4 | 4 | 1 | 1 | 1 | 0 |
|  |  | S5 | 1 | 1 | 0 | 1 | 0 |
|  |  | **Mean** | 1,6 | 0,6 | 0,2 | 0,6 | 0,2 |
|  | BLOCK 4 | S1 | 1 | 0 | 0 | 0 | 0 |
|  |  | S2 | 1 | 0 | 1 | 0 | 1 |
|  |  | S3 | 0 | 2 | 0 | 0 | 1 |
|  |  | S4 | 3 | 0 | 0 | 1 | 0 |
|  |  | S5 | 0 | 2 | 0 | 1 | 0 |
|  |  | **Mean** | 1 | 0,8 | 0,2 | 0,4 | 0,4 |
| CONTROL | BLOCK 1 | S1 | 0 | 0 | 2 | 3 | 2 |
|  |  | S2 | 2 | 3 | 1 | 0 | 1 |
|  |  | S3 | 1 | 3 | 4 | 0 | 3 |
|  |  | S4 | 1 | 0 | 0 | 2 | 0 |
|  |  | S5 | 1 | 2 | 1 | 2 | 1 |
|  |  | **Mean** | 1 | 1,6 | 1,6 | 1,4 | 1,4 |
|  | BLOCK 2 | S1 | 0 | 1 | 1 | 0 | 0 |
|  |  | S2 | 4 | 1 | 1 | 1 | 1 |
|  |  | S3 | 9 | 2 | 0 | 1 | 3 |
|  |  | S4 | 6 | 1 | 2 | 1 | 1 |
|  |  | S5 | 3 | 0 | 1 | 2 | 1 |
|  |  | **Mean** | 4,4 | 1 | 1 | 1 | 1,2 |
|  | BLOCK 3 | S1 | 1 | 0 | 3 | 0 | 1 |
|  |  | S2 | 0 | 2 | 1 | 2 | 1 |
|  |  | S3 | 0 | 1 | 0 | 2 | 2 |
|  |  | S4 | 5 | 4 | 2 | 1 | 0 |
|  |  | S5 | 1 | 0 | 1 | 0 | 0 |
|  |  | **Mean** | 1,4 | 1,4 | 1,4 | 1 | 0,8 |
|  | BLOCK 4 | S1 | 0 | 1 | 0 | 0 | 0 |
|  |  | S2 | 3 | 0 | 0 | 0 | 0 |
|  |  | S3 | 0 | 1 | 0 | 0 | 0 |
|  |  | S4 | 0 | 2 | 0 | 0 | 0 |
|  |  | S5 | 1 | 0 | 2 | 0 | 0 |
|  |  | **Mean** | 0,8 | 0,8 | 0,4 | 0 | 0 |

**SUPPLEMENTARY MATERIAL TABLE S3** | Evolution of number of *N. campestris* adults captured in spring 2023 during the test period after spraying with *M. brunneum* EAMa 01/58-Su strain. Fungi were applied in treatment plots on the first day of sampling in each year and treatment blocks.

|  |  | Sampling date | 03/04/2023 | 05/04/2023 | 07/04/2023 | 09/04/2023 | 11/04/2023 |
| --- | --- | --- | --- | --- | --- | --- | --- |
| TREATMENT | BLOCK 1 | M1 | 1 | 2 | 12 | 7 | 0 |
|  |  | M2 | 6 | 6 | 4 | 4 | 4 |
|  |  | M3 | 7 | 3 | 4 | 13 | 9 |
|  |  | **TOTAL** | 14 | 11 | 20 | 24 | 13 |
|  | BLOCK 2 | M1 | 0 | 1 | 1 | 0 | 0 |
|  |  | M2 | 0 | 0 | 4 | 1 | 0 |
|  |  | M3 | 0 | 0 | 0 | 0 | 1 |
|  |  | **TOTAL** | 0 | 1 | 5 | 1 | 1 |
|  | BLOCK 3 | M1 | 1 | 0 | 0 | 0 | 0 |
|  |  | M2 | 1 | 1 | 1 | 0 | 1 |
|  |  | M3 | 1 | 0 | 0 | 3 | 1 |
|  |  | **TOTAL** | 3 | 1 | 1 | 3 | 2 |
|  | BLOCK 4 | M1 | 0 | 1 | 1 | 2 | 1 |
|  |  | M2 | 1 | 0 | 0 | 1 | 0 |
|  |  | M3 | 2 | 1 | 3 | 0 | 4 |
|  |  | **TOTAL** | 3 | 2 | 4 | 3 | 5 |
| CONTROL | BLOCK 1 | M1 | 0 | 4 | 2 | 2 | 3 |
|  |  | M2 | 0 | 0 | 1 | 7 | 5 |
|  |  | M3 | 0 | 2 | 5 | 7 | 12 |
|  |  | **TOTAL** | 0 | 6 | 8 | 16 | 20 |
|  | BLOCK 2 | M1 | 3 | 2 | 9 | 15 | 19 |
|  |  | M2 | 6 | 7 | 15 | 16 | 11 |
|  |  | M3 | 2 | 13 | 7 | 7 | 8 |
|  |  | **TOTAL** | 11 | 22 | 31 | 38 | 38 |
|  | BLOCK 3 | M1 | 0 | 0 | 3 | 0 | 0 |
|  |  | M2 | 0 | 0 | 0 | 2 | 1 |
|  |  | M3 | 4 | 1 | 4 | 3 | 5 |
|  |  | **TOTAL** | 4 | 1 | 7 | 5 | 6 |
|  | BLOCK 4 | M1 | 0 | 0 | 3 | 3 | 4 |
|  |  | M2 | 1 | 0 | 5 | 3 | 4 |
|  |  | M3 | 2 | 0 | 1 | 2 | 1 |
|  |  | **TOTAL** | 3 | 0 | 9 | 8 | 9 |

**SUPPLEMENTARY MATERIAL TABLE S4** | Evolution of number of *N. campestris* adults captured in spring 2024 during the test period after spraying with *M. brunneum* EAMa 01/58-Su strain. Fungi were applied in treatment plots on the first day of sampling in each year and treatment blocks.

|  |  | Sampling Date | 08/04/2024 | 10/04/2024 | 12/04/2024 | 14/04/2024 | 16/04/2024 |
| --- | --- | --- | --- | --- | --- | --- | --- |
| TREATMENT | BLOCK 1 | M1 | 9 | 25 | 20 | 10 | 2 |
|  |  | M2 | 27 | 18 | 22 | 12 | 4 |
|  |  | M3 | 38 | 22 | 32 | 5 | 9 |
|  |  | **TOTAL** | 74 | 65 | 74 | 27 | 15 |
|  | BLOCK 2 | M1 | 10 | 3 | 14 | 16 | 6 |
|  |  | M2 | 15 | 22 | 12 | 14 | 2 |
|  |  | M3 | 46 | 29 | 29 | 4 | 10 |
|  |  | **TOTAL** | 71 | 54 | 55 | 34 | 18 |
|  | BLOCK 3 | M1 | 2 | 1 | 2 | 2 | 0 |
|  |  | M2 | 2 | 2 | 0 | 0 | 1 |
|  |  | M3 | 2 | 0 | 0 | 1 | 0 |
|  |  | **TOTAL** | 6 | 3 | 2 | 3 | 1 |
|  | BLOCK 4 | M1 | 1 | 1 | 3 | 0 | 1 |
|  |  | M2 | 2 | 1 | 2 | 2 | 2 |
|  |  | M3 | 3 | 1 | 2 | 2 | 1 |
|  |  | **TOTAL** | 6 | 3 | 7 | 4 | 4 |
| CONTROL | BLOCK 1 | M1 | 1 | 4 | 14 | 12 | 4 |
|  |  | M2 | 8 | 16 | 23 | 6 | 11 |
|  |  | M3 | 4 | 31 | 14 | 21 | 19 |
|  |  | **TOTAL** | 13 | 51 | 51 | 39 | 34 |
|  | BLOCK 2 | M1 | 10 | 10 | 27 | 19 | 12 |
|  |  | M2 | 36 | 23 | 33 | 9 | 11 |
|  |  | M3 | 18 | 26 | 14 | 25 | 23 |
|  |  | **TOTAL** | 64 | 59 | 74 | 53 | 46 |
|  | BLOCK 3 | M1 | 14 | 8 | 18 | 3 | 6 |
|  |  | M2 | 1 | 10 | 5 | 11 | 14 |
|  |  | M3 | 7 | 24 | 13 | 5 | 5 |
|  |  | **TOTAL** | 22 | 42 | 36 | 19 | 25 |
|  | BLOCK 4 | M1 | 2 | 1 | 1 | 0 | 3 |
|  |  | M2 | 1 | 3 | 3 | 2 | 1 |
|  |  | M3 | 1 | 1 | 1 | 2 | 1 |
|  |  | **TOTAL** | 4 | 5 | 5 | 4 | 5 |
